# Angiogenic regulatory influence of extracellular matrix deposited by resting state asthmatic and non‐asthmatic airway smooth muscle cells is similar

**DOI:** 10.1111/jcmm.16648

**Published:** 2021-06-18

**Authors:** Alen Faiz, Louise M. Harkness, Gavin Tjin, Victor Bernal, Peter Horvatovich, Alan James, John G. Elliot, Janette K. Burgess, Anthony W. Ashton

**Affiliations:** ^1^ Respiratory Cellular and Molecular Biology Woolcock Institute of Medical Research. Sydney NSW Australia; ^2^ Emphysema Center Woolcock Institute of Medical Research The University of Sydney Glebe NSW Australia; ^3^ Respiratory Bioinformatics and Molecular Biology Faculty of Science University of Technology Sydney Ultimo NSW Australia; ^4^ Department of Pathology and Medical Biology Groningen Research Institute for Asthma and COPD University Medical Center Groningen University of Groningen Groningen The Netherlands; ^5^ Department of Pulmonology University Medical Center Groningen University of Groningen Groningen The Netherlands; ^6^ Discipline of Pharmacology School of Medical Sciences The University of Sydney Sydney NSW Australia; ^7^ Central Clinical School The University of Sydney Sydney NSW Australia; ^8^ Bernoulli Institute (BI) University of Groningen Groningen The Netherlands; ^9^ Department of Pharmacy Analytical Biochemistry University of Groningen Groningen The Netherlands; ^10^ Department of Pulmonary Physiology and Sleep Medicine West Australian Sleep Disorders Research Institute Sir Charles Gairdner Hospital Perth WA Australia; ^11^ School of Medicine and Pharmacology University of Western Australia Perth WA Australia; ^12^ Department of Pathology and Medical Biology KOLFF Institute University Medical Center Groningen University of Groningen Groningen The Netherlands; ^13^ Division of Perinatal Research Kolling Institute of Medical Research Sydney NSW Australia; ^14^ Present address: St Vincent’s Institute Medical Research Fitzroy Vic. Australia

**Keywords:** airway smooth muscle, angiogenesis, asthma, extracellular matrix, inflammation

## Abstract

The extracellular matrix (ECM) is the tissue microenvironment that regulates the characteristics of stromal and systemic cells to control processes such as inflammation and angiogenesis. Despite ongoing anti‐inflammatory treatment, low levels of inflammation exist in the airways in asthma, which alters ECM deposition by airway smooth muscle (ASM) cells. The altered ECM causes aberrant behaviour of cells, such as endothelial cells, in the airway tissue. We therefore sought to characterize the composition and angiogenic potential of the ECM deposited by asthmatic and non‐asthmatic ASM. After 72 hours under non‐stimulated conditions, the ECM deposited by primary human asthmatic ASM cells was equal in total protein, collagen I, III and fibronectin content to that from non‐asthmatic ASM cells. Further, the matrices of non‐asthmatic and asthmatic ASM cells were equivalent in regulating the growth, activity, attachment and migration of primary human umbilical vein endothelial cells (HUVECs). Under basal conditions, asthmatic and non‐asthmatic ASM cells intrinsically deposit an ECM of equivalent composition and angiogenic potential. Previous findings indicate that dysregulation of the airway ECM is driven even by low levels of inflammatory provocation. This study suggests the need for more effective anti‐inflammatory therapies in asthma to maintain the airway ECM and regulate ECM‐mediated aberrant angiogenesis.

## 1 INTRODUCTION

The underlying pathophysiology of asthma includes chronic airway inflammation, reversible obstruction, hypersensitive bronchial constriction and altered vascularization of the airway wall.^1^ Despite effective symptom‐controlling therapies, asthma continues to affect the lives of 334 million individuals worldwide^2^ and leads to 3630 deaths each year in the USA alone.^3^ The structural abnormalities of the asthmatic airway, collectively referred to as airway remodelling,^4^, ^5^ have been hypothesized to contribute to the development of asthma and its severity.^6^, ^7^, ^8^, ^9^ This study focuses on two characteristic features of airway remodelling in asthma, the altered extracellular matrix (ECM) and excessive vascularization.

The airways of patients with asthma have alterations in the composition and functionality of the ECM.^10^, ^11^, ^12^ As a bioactive network of proteins, the ECM is vital for tissue structure and integrity^13^ and is the microenvironment which regulates cell growth, metabolism, attachment and movement.^14^, ^15^ The ECM deposited by ASM cells contributes to the protein microenvironment of the muscle bundles, sub‐epithelial space and adventitia. The bronchial circulatory networks are situated in the sub‐epithelial space and adventitia, running alongside the ASM bundles. A number of groups have suggested that the altered pattern of proteins within the asthmatic airway ECM, such as increased pro‐angiogenic proteins fibronectin and collagen I,^16^, ^17^ could drive blood vessel growth.^1^ Endothelial cells entering the asthmatic airway tissue come into direct contact with the altered pro‐angiogenic ECM microenvironment and are stimulated to proliferate and extend the vascular bed in an unregulated manner. To date, there have been no studies exploring the angiogenic potential of the asthmatic airway ECM. It is unclear what the exact nature of the composition and functionality of the ECM deposited by asthmatic ASM cells would be under non‐stimulatory conditions and whether abnormal ECM deposition is an intrinsic feature of asthmatic ASM.

This study sought to investigate the gene expression pattern of asthmatic ASM cells in the absence of stimulation, as well as the composition and functionality of the ECM they deposit. Our aim was to determine whether the angiogenic potential of the asthmatic ASM‐ECM was different from that of the non‐asthmatic ASM by assessing the ability of the matrices to regulate endothelial cell behaviour.

## 2 MATERIALS AND METHODS

The supplier details of all materials used in this manuscript and full methodological details are provided in the Online Supplement.

### 2.1 Study population

Endobronchial biopsies or explanted tissue were obtained from 36 individuals with doctor‐diagnosed asthma. Airway remodelling was evident in a subset of these asthmatic patients, as previously described.^18^ Lung tissue was obtained from 34 non‐asthmatics (bronchoscopies from healthy volunteers, explanted lung tissue from healthy donors or “macroscopically normal” tissue from resected lung tissue of carcinoma patients). Full details are provided in the Table S1.

### 2.2 Ethics approval

Written informed consent was provided by individuals undergoing scheduled lung resection, lung transplantation or bronchoscopy. A hospital pathologist identified and supplied all “macroscopically normal” tissue. The Ethics Review Committees of the South West Sydney Area Health Service, Royal Prince Alfred Hospital, Macquarie University and the University of Sydney and Sir Charles Gairdner Group Human Research Ethics Committees provided approval for this study (Human Research Ethics Committee Approval numbers AU/1/76B9015—Approved 14/11/2011, X14‐0045—Approved 07/04/2014, 5201300355—Approved 29/05/2015, 11507—Approved 25/10/2011 and 10139—Approved 28/10/2011, HREC No:2015‐053). For the collection of human umbilical cords, informed consent was obtained from pregnant women prior to delivery by caesarean section. Ethics approval was provided by the Northern Sydney Local Health District (1004‐145M—Approved 05/05/2010).

### 2.3 Isolation and culture of primary human ASM cells and human umbilical vein endothelial cells

Human ASM cells were isolated by macrodissection as previously described^19^, ^20^, ^21^ and used between passages 2 and 6. The experiments for which cells from each donor were used are described in Table S1. Human umbilical vein endothelial cells (HUVECs) were isolated as previously described^22^ and used at passages 1‐5. All cells tested negative for the presence of Mycoplasma before use.

### 2.4 Gene expression patterns of unstimulated non‐asthmatic and asthmatic ASM cells *in* *vitro*

mRNA was isolated from unstimulated non‐asthmatic and asthmatic ASM cells before reverse transcription which was performed using MML‐V. cDNA was collected from ASM cells of three non‐asthmatic and three asthmatic patients, grown under non‐stimulatory conditions (0.1% BSA in quiescing media) for 72 hours. The samples were pooled to create a single non‐asthmatic and asthmatic sample, which were loaded onto a Taqman^®^ RT‐PCR array for Human ECM & Adhesion Molecules (#4414133). A second TaqMan^®^ RT‐PCR array for Human Angiogenesis (#4414071) was performed using pooled ASM cDNA from 7 non‐asthmatic and 8 asthmatic patients. Relative abundance of gene expression was calculated using the ∆ cycle threshold method^23^ and normalized to two housekeeping genes (18S and GAPDH). In a max difference analysis, a fold change of > ±2 of asthmatic vs non‐asthmatic ASM cells was considered as a true difference. A selection of individual genes found to be differentially expressed on the RT‐PCR arrays was validated in individual donor samples using qPCR, Figure S1 in the online supplement.

### 2.5 Comparative GO term enrichment

A gene ontology (GO) enrichment analysis was performed with the R package g ProfileR version 0.6.4.^24^ This package applied a correction for multiple tests designed for ontology analysis by default (g:SCS).^24^ The enrichment of the genes under study was contrasted against the enrichment obtained for a randomly assembled group of 121 genes. Further details about this strategy are provided in the online supplement.

### 2.6 ASM‐ECM deposition and decellularization

Non‐asthmatic and asthmatic ASM cells were seeded onto tissue culture surfaces at a density of 1 × 10^4^ cells/cm^2^ and expanded in 10% FBS‐DMEM culture media for 72 hours. After synchronization for 48 hours, the ASM cells were immersed in fresh 0.1% (w/v) BSA quiescing media for 24 hours,^19^, ^20^ before the ASM cell‐deposited ECM was harvested as described previously.^15^

### 2.7 Total protein content of the unstimulated ASM‐ECM

The total protein content in the ECM from non‐asthmatic and asthmatic ASM cells was measured using a BCA assay.

### 2.8 Collagen and fibronectin in the unstimulated ASM cell deposited‐ECM

In the decellularized ASM‐ECM, deposited collagen I and III content was assessed using picosirius red staining,^25^ and deposited fibronectin was quantified using a solid‐phase ELISA ^26^ as previously described. Collagen I, III and fibronectin were each quantified as the % of total ECM protein.

### 2.9 Collagen I fibre organization within the ASM bundles of non‐asthmatic and asthmatic airway sections

Collagen I fibre organization within the ASM bundles of non‐asthmatic and asthmatic airway tissue was examined by second harmonic generation (SHG) signal detection as previously described.^27^, ^28^ Forward and backward SHG signals within three randomly selected regions within the ASM bundles were detected and quantified as previously described,^27^ and the signal intensity was quantified using Fiji software.^29^

### 2.10 The functional properties of ECM from unstimulated ASM cells: HUVEC behaviour on decellularized ASM‐ECM

After quiescing for 36 hours in supplement‐free HUVEC culture media, HUVECs were seeded onto decellularized ECM derived from non‐asthmatic and asthmatic ASM cells in fresh supplement‐free HUVEC culture media. HUVEC proliferation and metabolic activity were assessed 72 hours after seeding using CyQUANT and thaizolyl blue tetrazolium bromide (MTT) assays. Attachment of HUVECs to ASM‐ECM was performed at 37°C for 30 minutes and quantified with toluidine blue staining as previously described.^30^ HUVEC proliferation, metabolic activity and attachment to the ASM‐ECM were normalized to HUVEC responses on wells without ASM‐ECM. HUVEC chemotaxis was performed in a transwell system (8 μm pore size) using a 10 ng/mL VEGF‐A chemoattractant, as previously described.^31^ Migrating HUVECs were fixed, stained and mounted and five regions on each membrane were imaged and analysed.

### 2.11 Statistical analyses

Data analyses were performed using GraphPad Prism software (version 6.0, GraphPad Software). All the data were expressed as mean ± standard error of the mean (SEM). Data were compared using unpaired *t* tests and one‐way ANOVA using a Bonferroni post‐test where data fitted a Gaussian distribution. Mann‐Whitney tests were used for non‐parametric data analyses. Outliers were identified using Grubb’s test and were sequentially excluded from the respective data sets. Groups showing *P* ≤ .05 were considered significantly different.

## 3 RESULTS

### 3.1 Non‐asthmatic and asthmatic ASM cells have differential basal gene expression patterns

**FIGURE 1:**
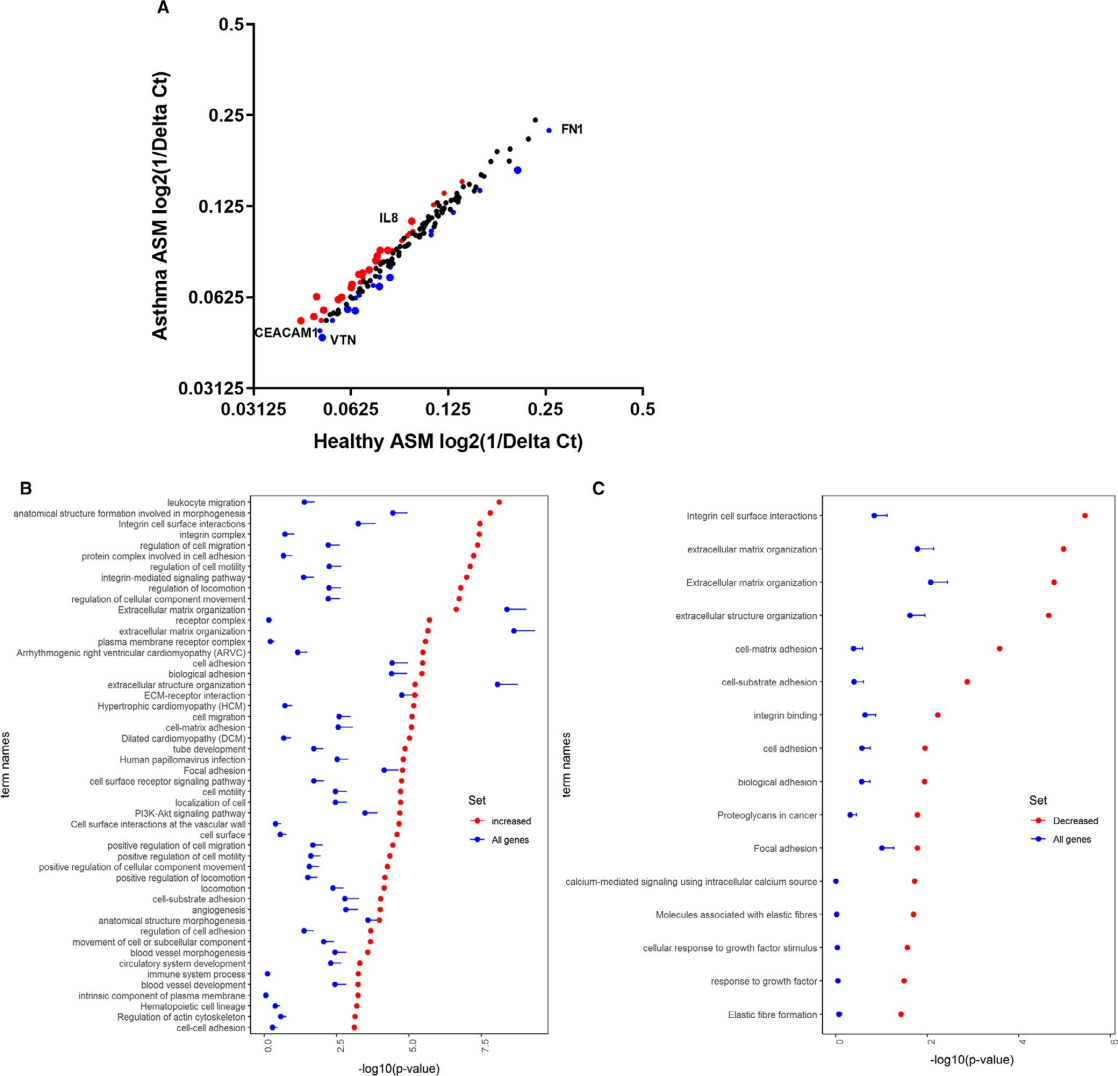
Gene expression of unstimulated non‐asthmatic and asthmatic ASM cells in vitro. A, Max difference plot of data from RT‐PCR arrays conducted on non‐asthmatic and asthmatic ASM cells in vitro, red dots 17 up‐regulated genes, blue dots 6 down‐regulated genes. B, Top 50 enriched GOs from the increased genes, C, enriched GOs from the decreased in asthmatic ASM cells. In (B) and (C), the red dots show the –log_10_ *P*‐value of the enriched terms retrieved for the 17 increased and for the six decreased gene sets, respectively. The blue dots show the average –log_10_ *P*‐value of the same terms obtained from 100 random samples of genes (of the same size (ie 17 and 2 genes, respectively). The error bars of the blue dots show standard errors of the mean –log_10_ *P*‐value. Abbreviations: A, asthmatic; ASM, airway smooth muscle; Ct, cycle threshold; FDR, false discovery rate; GO, gene ontology; NA, non‐asthmatic; NCBI, National Center for Biotechnology Information

Differences in the expression of angiogenic and adhesion molecule genes between unstimulated non‐asthmatic and asthmatic ASM cells, relative to 18S and GAPDH, are summarized in Tables S2 and S3. The expression of three genes were validated using individual donor samples by qRT‐PCR (Figure S1). The max difference analyses identified 17 genes with increased expression and 6 genes with decreased expression in asthmatic ASM cells compared with non‐asthmatic ASM cells (Figure 1A). GO term enrichment analyses identified the increased genes in the asthmatic ASM cells as associated with “leukocyte migration”, “anatomical structure function involved in morphogenesis” and “integrin cell surface interactions”, while the decreased genes were associated with “integrin cell surface interactions” and “extracellular matrix organization” (Figure 1B,C, Tables S2 and S3).

### 3.2 The composition and profile of ECM deposited by unstimulated non‐asthmatic and asthmatic ASM cells is not different

**FIGURE 2:**
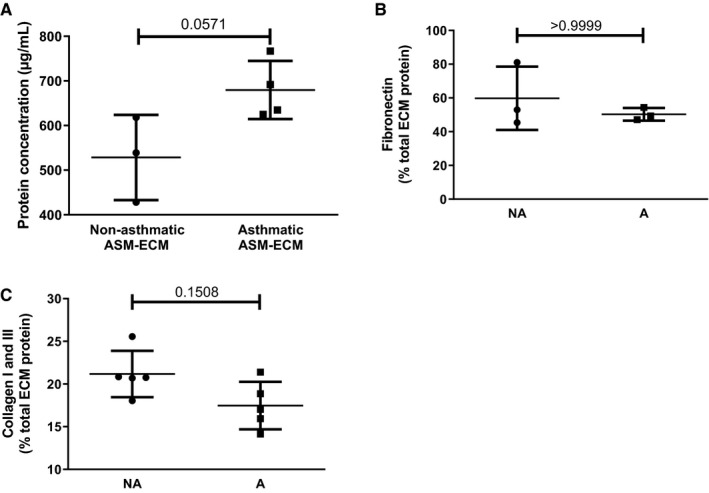
The ECM deposited by unstimulated non‐asthmatic and asthmatic ASM cells in vitro is similar in protein mass and composition. A, Total amount of protein deposited into the ECM by unstimulated non‐asthmatic (● ;N = 3) and asthmatic ASM cells (⬛;N = 4) without stimulation was measured using a BCA assay. B, Fibronectin content in the non‐asthmatic and asthmatic ASM‐ECM was determined using a solid‐phase ELISA (N = 3 for both). C, Collagen I and III in the decellularized ASM‐ECM was measured by picosirius red staining (N = 5 for both). Data are presented as either protein concentration (µg/mL) or mean ± SEM % total ECM protein. Groups were compared using a Mann‐Whitney test. Grubb’s test was used to identify outliers which were sequentially excluded. Abbreviations: A, asthmatic; BCA, bicinchoninic acid; NA, non‐asthmatic; ns, non‐significant, *P*, probability, SEM, standard error of the mean

Unstimulated asthmatic and non‐asthmatic ASM cells deposited a similar amount of total protein in the ECM (non‐asthmatic ASM‐ECM: 528.4 ± 55.1 µg/mL [N = 3]; asthmatic ASM‐ECM: 679.6 ± 32.6 µg/mL [N = 4]) (Figure 2A). Comparable amounts of fibronectin were deposited into the ECM of the non‐asthmatic and asthmatic ASM (non‐asthmatic ASM‐ECM: 59.8 ± 1.2% total ECM protein; asthmatic ASM‐ECM: 50.3 ± 1.2%; N = 3 for both) (Figure 2B). Similarly, equivalent amounts of collagen I and III were deposited (non‐asthmatic ASM‐ECM: 21.2 ± 2.7% total ECM protein; asthmatic ASM‐ECM: 17.5 ± 2.8%; N = 5 for both) (Figure 2C).

**FIGURE 3:**
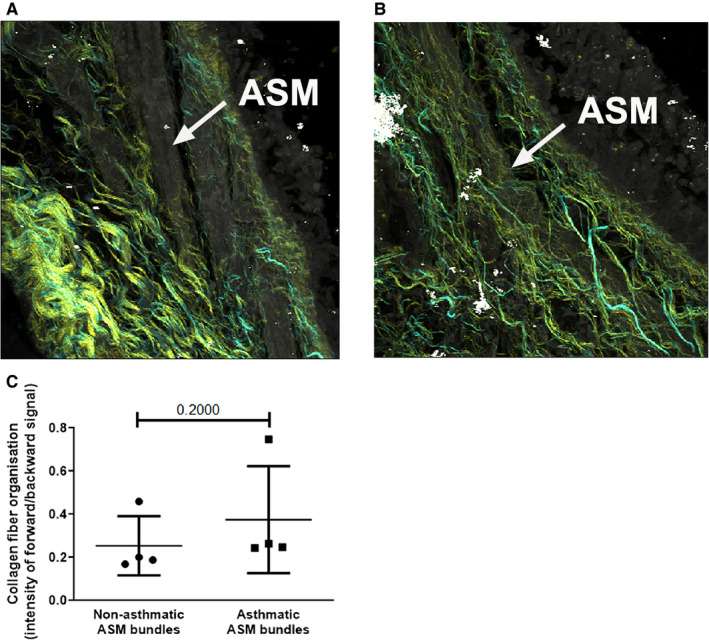
The organization of collagen I fibres within the ASM bundles in the airways of non‐asthmatic or asthmatic individuals ex vivo. The arrangement of collagen I in the ASM bundles in the (A) non‐asthmatic (N = 4) and (B) asthmatic (N = 4) airways was assessed by SHG. C, The SHG signal was quantified. Differences between the groups were determined with a Mann‐Whitney test. Abbreviations: ASM, airway smooth muscle; SHG, second harmonic generation E, epithelium; L, lumen; *P*, probability

The organization of the collagen fibres within the ASM bundles in airway tissues in vivo was similar in asthmatic and non‐asthmatic patients (non‐asthmatic: 0.25 ± 0.07 ratio of organized to disorganized collagen units; asthmatic: 0.38 ± 0.12; N = 4&4) (Figure 3), indicating that the collagen crosslinking, and thus fibre maturity, was not altered in the vicinity of the ASM bundles in the asthmatic airway. These data indicate that the basal differential gene expression does not translate into changes in protein structure in the immediate vicinity of the asthmatic ASM.

### 3.3 HUVECs behave similarly in response to the ECM from unstimulated non‐asthmatic and asthmatic ASM

**FIGURE 4:**
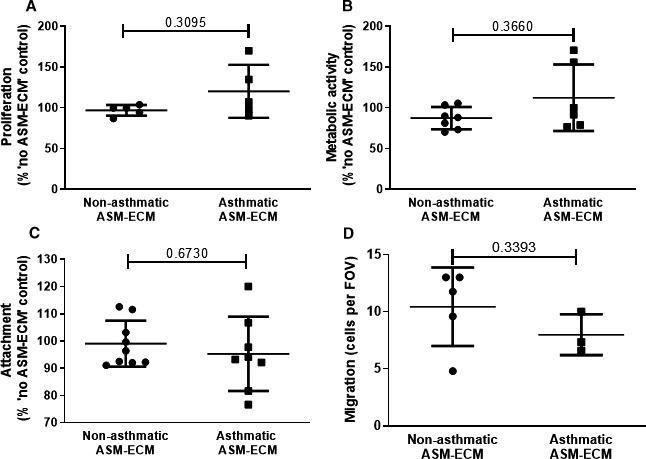
No difference in HUVEC behaviour was seen on non‐asthmatic or asthmatic ASM‐ECM. A, HUVEC proliferation after 72 hours on a decellularized non‐asthmatic ASM‐ECM (●) or asthmatic ASM‐ECM (⬛; N = 5 for both) was quantified with a CyQUANT assay. B, HUVEC metabolic activity after 72 hours on the non‐asthmatic ASM‐ECM (N = 7) and asthma ASM‐ECM (N = 6) was quantified using an MTT assay. C, HUVEC attachment to the non‐asthmatic (N = 9) and asthmatic (N = 8) ASM‐ECM after 30 minutes was quantified with toluidine blue staining. D, HUVEC chemotaxis to 10ng/mL VEGF‐A through membrane coated with the non‐asthmatic (N = 4) and asthmatic (N = 3) ASM‐ECM was assessed with toluidine blue staining and manual cell counts. HUVEC function was quantified as % “no ASM cell” or cells per FOV and presented as mean ± SEM. Differences between the groups were determined with Mann‐Whitney tests. Grubb's test was used to identify outliers which were sequentially excluded. Abbreviations: A, asthmatic; FOV, field of view; MTT, thaizolyl blue tetrazolium bromide; NA, non‐asthmatic; ns, non‐significant; *P*, probability; SEM, standard error of the mean; VEGF‐A, vascular endothelial growth factor‐A

On the decellularized ECM from non‐asthmatic and asthmatic ASM, there was no difference in HUVEC proliferation (non‐asthmatic ASM‐ECM: 97.0 ± 3.0% “no ASM‐ECM”; asthmatic ASM‐ECM: 120.2 ± 14.5%; N = 5&5) (Figure 4A), nor HUVEC metabolic activity (non‐asthmatic ASM‐ECM: 87.5 ± 5.2% “no ASM‐ECM” [N = 7]; asthmatic ASM‐ECM: 112.4 ± 16.6% [N = 6] (Figure 4B). HUVEC attachment to the non‐asthmatic and asthmatic ASM‐ECM was also similar (non‐asthmatic ASM‐ECM: 99.1 ± 2.8% “no ASM‐ECM” control [N = 9]; asthmatic ASM‐ECM: 95.3 ± 4.8% [N = 8]) (Figure 4C). HUVEC chemotaxis through the non‐asthmatic and asthmatic ASM‐ECMs was similar (non‐asthmatic ASM‐ECM: 9.8 ± 1.8 cell per field of view, [N = 4]; asthmatic ASM‐ECM: 8.0 ± 1.0 [N = 3]) (Figure 4D) and migration through both matrices were reduced compared with a fibronectin matrix (fibronectin matrix: 20.5 ± 3.4 [N = 3]; compared with non‐asthmatic ASM‐ECM *P* = .03 and asthmatic ASM‐ECM *P* = .02) (data not presented).

Overall, there was no difference observed in HUVEC cell behaviour measured on the decellularized ECM from unstimulated non‐asthmatic and asthmatic ASM.

## 4 DISCUSSION

Under non‐stimulatory conditions asthmatic ASM cells deposit an ECM which is similar in composition and functionality to that deposited by non‐asthmatic ASM cells. These data, in light of the current literature, suggest the asthmatic ASM cells may be depositing an irregular ECM in response to the stimulatory conditions of the inflamed airway, and highlight the interplay between airway inflammation and airway remodelling in asthma.

It has been long accepted that the asthmatic airway ECM, contributed to by the ASM cells, is altered in amount, composition and functionality.^15^, ^32^ Importantly, these changes contribute significantly to airway remodelling,^33^ including alterations in the vascular compartment in the asthmatic airways. However, these previous studies have examined the composition and functionality of the ASM‐ECM in the context of chronic inflammation, either using pro‐inflammatory stimuli to activate ASM cells to deposit ECM in vitro or examining tissue ECM of samples extracted from asthmatic airways ex vivo.^12^, ^15^, ^34^, ^35^ While experimentally valuable to assess the ECM in the complex milieu in the asthmatic airway, including under volatile conditions of airway inflammation, introducing these conditions to an in vitro model adds a complex variable which makes it difficult to pinpoint the origin of the constitutively “abnormal” ECM. The results of this study demonstrated the asthmatic and non‐asthmatic ASM deposit an ECM that is equal in the quantity of total protein, fibronectin, and collagen I and III. This study is the first to suggest that under non‐stimulatory conditions asthmatic ASM cells deposit an ECM that reflects that of non‐asthmatic ASM cells. The ECM from asthmatic ASM cells also facilitates the behaviour of endothelial cells in a similar manner to the non‐asthmatic ASM cell‐ECM, suggesting that the ASM‐ECM maintains stability of the angiogenic process. The results of this study suggest that asthmatic ASM cells are intrinsically different from non‐asthmatic ASM cells at the gene level, however, stimuli such as those present during chronic inflammation are required for predisposed differences to manifest into the ECM abnormalities extensively described in the literature.^15^, ^35^ Under stimulatory conditions in previous in vitro studies, the asthmatic ASM deposit an irregular ECM, such as increased collagen I under serum‐rich conditions,^15^, ^36^ and increased collagen I, III and fibronectin under stimuli modelling inhaled cigarette smoke.^37^ Likewise, ex vivo, the asthmatic airway tissue is reported to contain increased amounts of ECM proteins, including collagen^10^, ^11^, ^35^, ^38^

The findings of this current study agree with previous reports of ASM cells expressing increased amounts of hyaluronic acid under serum‐stimulation which was suppressed when the serum stimulation was reduced,^39^ and reports of no disproportional increase of ECM proteins within the ASM layer of asthmatic individuals compared with healthy volunteers.^40^

While the literature has previously reported on the collagen levels, in the static state in the asthmatic tissue, this study was the first to assess the organization of collagen fibres within the ASM bundles in the asthmatic airway. The organization of the collagen fibres allows for a deeper examination of the remodelling process within the tissue. Poorly organized collagen structure may represent newly formed fibres, while mature collagen fibres are more organized in structure. A significantly greater amount of immature collagen I fibres have recently been reported in the airway tissue of subjects with chronic obstructive pulmonary disease.^27^ Surprisingly, despite the increase in static amounts of collagen previously reported in the asthmatic airway, our study found that the organization of the collagen filaments within the ASM bundles did not differ from that seen in the airways of non‐asthmatic individuals. This was in contrast to previous reports of disorganized fibrillar collagen within large and small asthmatic airways. Mostaco‐Guidolin et al focused on fibroblast packaging of collagen fibrils, which was aberrant in asthma, suggesting possible differences in ECM organization by ASM and fibroblasts in asthmatic airways.^41^

To date, there have been no studies examining the angiogenic potential of the asthmatic airway ECM. It has been proposed that the alterations to the ECM reported in the asthmatic airway tissue establish an environment which facilitates endothelial cell activity and thus contributes to the aberrant angiogenesis of the remodelled airway.^42^ The current study showed the ECM of asthmatic ASM cells deposited under basal conditions did not differentially affect endothelial cell proliferation, metabolic activity, attachment and migration, and thus did not impact the processes involved in formation and movement of blood vessel sprouting tips during the early stages of angiogenesis. As the sprouting tip travels through the localized tissue, the endothelial cells come into direct contact with the ECM microenvironment, influencing endothelial cell growth rates, survival and movement, thus impacting blood vessel formation. The findings of this study reveal the ECM deposited by asthmatic ASM cells under non‐stimulatory conditions does not induce irregular behaviour of endothelial cells entering this microenvironment.

Our findings indicate that when all stimulation is removed the ECM deposited by asthmatic ASM cells functions as a healthy matrix which maintains controlled HUVEC growth and vascular expansion.

### 4.1 Limitations of this study

We acknowledge there are limitations to our study findings. The small sample size in the in vitro studies limited the interpretation of the data presented at the population level. Regardless, the value of the study lies in the use of primary human cells rather than immortalized cell lines, as these best reflect the cells of the human lung thereby increasing the translation of our data towards human disease. We also acknowledge the limitations related to the use of HUVECs, instead of adult primary human lung endothelial cells. HUVECs, despite their foetal origin, largely reproduce the (patho)physiology of the vascular tree in adults (as defined by ref. [43]) and, when used at low passage (as we have in this study), are a faithful surrogate for pulmonary endothelial cells.^44^ HUVECs, despite being macrovascular in origin recapitulate the angiogenic phenotype of mature blood vessels (including VEGF responsiveness, adhesion molecule expression (such as E‐selectin, PECAM‐1 and cadherin‐5) and the synthesis of extracellular proteins).^45^ The use of HUVEC pooled from 4 to 5 donors negates the impact of gender and inter‐donor variability on outcomes of endothelial behaviour that have previously been reported.^45^ As such, we believe that the low passage HUVEC used in this study represent a readily accessible, endothelial cell type with authentic, reproducible responses to angiogenic stimuli such as the ECM of ASM cells from non‐asthmatic and asthmatic donors as used in this study.

### 4.2 Implications of this study

This study provides further evidence of the interplay between airway inflammation and airway remodelling, showing the airway wall ECM composition and bioactive properties are likely to be highly sensitive to the chronic airway inflammatory conditions in asthma. There is debate over the limited effectiveness of current anti‐inflammatory treatments, which do not completely resolve airway inflammation, on airway remodelling.^18^, ^34^ The residual airway inflammation remaining in treated asthmatics may be enough to induce ASM to deposit an altered ECM. As the ECM is a central component of the airway tissue,^46^ the generation of an abnormal ECM in response to pro‐inflammatory conditions has a flow on effect for the persistence and progression of the cell dysregulation in the airway^47^, ^48^ and airway remodelling, particularly aberrant vascularization.^49^ The current study adds a new dimension to the urgency to ablate airway inflammation in asthma, and the need to develop anti‐remodelling agents targeted at the central ECM as add‐on therapy to existing asthma treatments.

## CONFLICT OF INTEREST

The authors declare that they have no conflict of interest related to this study.

## AUTHOR CONTRIBUTION

**Alen Faiz:** Conceptualization (equal); Data curation (equal); Formal analysis (equal); Investigation (equal); Methodology (equal); Validation (equal); Visualization (equal); Writing‐original draft (equal); Writing‐review & editing (equal). **Louise Margaret Harkness:** Conceptualization (equal); Data curation (equal); Formal analysis (equal); Investigation (equal); Methodology (equal); Project administration (equal); Validation (equal); Visualization (equal); Writing‐original draft (equal); Writing‐review & editing (equal). **Gavin Tjin:** Data curation (equal); Formal analysis (equal); Investigation (equal); Methodology (equal); Validation (equal); Visualization (equal); Writing‐review & editing (equal). **Victor Arzola Bernal:** Data curation (equal); Formal analysis (equal); Investigation (equal); Visualization (equal); Writing‐review & editing (equal). **Peter Horvatovich:** Data curation (equal); Formal analysis (equal); Investigation (equal); Methodology (equal); Validation (equal); Writing‐review & editing (equal). **Alan James:** Data curation (equal); Formal analysis (equal); Resources (equal); Validation (equal); Writing‐review & editing (equal). **John Elliot:** Data curation (equal); Formal analysis (equal); Resources (equal); Writing‐review & editing (equal). **Janette K Burgess:** Conceptualization (equal); Data curation (equal); Formal analysis (equal); Funding acquisition (equal); Project administration (equal); Supervision (equal); Writing‐original draft (equal); Writing‐review & editing (equal). **Anthony Ashton:** Conceptualization (equal); Data curation (equal); Formal analysis (equal); Funding acquisition (equal); Investigation (equal); Project administration (equal); Supervision (equal); Validation (equal); Writing‐original draft (equal); Writing‐review & editing (equal).

## Supporting information

Supplementary Material

## Data Availability

The data that support the findings of this study are available from the corresponding author upon reasonable request.
