# Supplementary material for: Angiogenic regulatory influence of extracellular matrix deposited by resting state asthmatic and non‐asthmatic airway smooth muscle cells is similar
Source: J Cell Mol Med. 2021 Jun 18;25(13):6438–47. doi: 10.1111/jcmm.16648 (PMC8256353; doi:10.1111/jcmm.16648)
Supplement: Supplementary file 1 — Supplementary Material [file JCMM-25-6438-s001.docx]

# Angiogenic regulatory influence of extracellular matrix deposited by resting state asthmatic and nonasthmatic airway smooth muscle cells is similar.

**Online Supplement**

Faiz, A.^1,2,3,4,5,6,7^, Harkness, L. M.^1,5,6^, Tjin, G.^1,6,7 #^, Bernal, V.^8,9^, Horvatovich, P.^9^, James, A.^10,11^, Elliot, J. G.10, Burgess, J. K.^1,4,5,6,,7,12^, Ashton, A. W.^13^

Faiz, A. and Harkness, L. M. should be considered joint first author. Burgess, J. K. and Ashton, A. W. should be considered joint senior author.

^1^ Respiratory Cellular and Molecular Biology, Woolcock Institute of Medical Research. Sydney, NSW, Australia

^2^ Emphysema Center, Woolcock Institute of Medical Research, The University of Sydney, Glebe, NSW, Australia;

^3^ Faculty of Science, University of Technology Sydney, Respiratory Bioinformatics and Molecular Biology, Ultimo, NSW, Australia

^4^ University of Groningen, University Medical Center Groningen, Department of Pathology and Medical Biology, Groningen Research Institute for Asthma and COPD, Groningen, The Netherlands

^5^ University of Groningen, University Medical Center Groningen, Department of Pulmonology Groningen, The Netherlands

^6^ Discipline of Pharmacology, School of Medical Sciences, The University of Sydney. Sydney, NSW, Australia

^7^ Central Clinical School, The University of Sydney, Sydney, NSW, Australia

^8^ University of Groningen, Bernoulli Institute (BI), Groningen, The Netherlands.

^9^ University of Groningen, Department of Pharmacy, Analytical Biochemistry, Groningen, The Netherlands.

^10^ Department of Pulmonary Physiology and Sleep Medicine, West Australian Sleep Disorders Research Institute, Sir Charles Gairdner Hospital. Perth, WA, Australia

^11^ School of Medicine and Pharmacology, University of Western Australia, WA, Australia

^12^ University of Groningen, University Medical Center Groningen, Department of Pathology and Medical Biology, KOLFF Institute, Groningen, The Netherlands.

^13^ Division of Perinatal Research, Kolling Institute of Medical Research, Sydney, NSW, Australia

# current affiliation St Vincent’s Institute Medical Research, Fitzroy, Victoria, Australia

### Corresponding author:

Prof Janette Burgess

University Medical Center Groningen

Department of Pathology and Medical Biology

Hanzeplein 1

[IPC EA11]

9713 GZ Groningen

The Netherlands

phone:+31-50-3618043 (secr) / +31-50-3610985

fax: +31-50-3619911

email: j.k.burgess@umcg.nl

# Materials and Methods

## Materials

## All materials were obtained from Thermo Fisher (Life Technologies, Invitrogen, and Applied Biosystems; Waltham, MA, USA) unless stated otherwise. BSA, L-glutamine, pooled AB human serum, ECGS, heparin, BCA assay kit, MTT, NH_4_OH, Tris-HCl, Triton X-100, glycerol, sodium deoxycholate, ethanol, gelatin, Hank’s balanced salts, PMSF, H_3_PO_4_, DPX, and toluidine blue were obtained from Sigma-Aldrich (Castle Hill, NSW, AUS). FBS was supplied by DKSH (Hallam, VIC, AUS) while BBE was obtained from Lonza (Basel, Switzerland). ISOLATE RNA mini kit, sensifast mastermix, PCR-grade H_2_O, and dNTP’s were from Bioline (Alexandria, NSW, AUS), and 8μm polycarbonate pore transwells from Corning Life Sciences (Tewksbury, MA, USA). Mouse anti-human plasma fibronectin c-terminal and protease inhibitor cocktail set III (EDTA-free) were obtained from Merck Millipore (Billerica, MA, USA), while rabbit anti-mouse Ig HRP from DAKO (North Sydney, NSW, AUS). Fibronectin from human plasma was obtained from BD Biosciences (Oxford, UK) and the random primer 6 from New England Biolabs (Ipswich, MA, USA). NaCl from PanReac AppliChem (Barcelona, Spain), and both SDS and boric acid were obtained from Amresco (Solon, OH, USA).

## Study Population

Endobronchial biopsies or explanted lung tissue were obtained from 36 individuals who answered yes to the question, “has a doctor ever told you that you have asthma?”. Airway remodeling was evident in a subset of these asthmatic patients as previously described by our group [1]. Lung tissue was obtained from 34 non-asthmatics (endobronchial biopsies from healthy volunteers, explanted lung tissue from healthy volunteers or ‘macroscopically normal’ tissues from resected lung tissue of carcinoma patients). Full details are provided in Table S1.

**Table S1. Study population.** Demographic information and medical history were collected from non-asthmatic and asthmatic subjects or volunteers consenting to lung tissue collection and cell isolation from scheduled lung resections, lung transplantations, or research bronchoscopies.

| **No.** | **Diagnosis** | **Gender (M/F)** | **Age (years)** | **Smoking history** | **Sample** | **FEV_1_** | **FVC** | **Experiment** |
| --- | --- | --- | --- | --- | --- | --- | --- | --- |
| 1 | Asthma | M | 25 | - | Biopsy | - | - | 8 |
| 2 | Asthma | M | 33 | - | Biopsy | - | - | 10 |
| 3 | Asthma | F | 18 | N | Bronchoscopy | 3.21L | 3.49L | 3, 8, 9 |
| 4 | Asthma | M | 20 | N | Bronchoscopy | 3.13L | 5.07L | 4 |
| 5 | Asthma | M | 20 | N | Bronchoscopy | 5.09L | 6.24L | 1, 2, 8 |
| 6 | Asthma | M | 25 | N | Bronchoscopy | 3.87L | 5.41L | 4 |
| 7 | Asthma | M | 23 | N | Bronchoscopy | 2.80L | 4.47L | 1, 2 |
| 8 | Asthma | M | 22 | N | Bronchoscopy | 3.80L | 5.16L | 5, 8, 9 |
| 9 | Asthma | M | 21 | N | Bronchoscopy | 4.45L | 5.00L | 1, 2, 8 |
| 10 | Asthma | M | 45 | N | Biopsy | 4.00L | 5.36L | 2 |
| 11 | Asthma | - | - | - | - | - | - | 4 |
| 12 | Asthma | M | 38 | N | Biopsy | 3.22L | 4.64L | 3 |
| 13 | Asthma | F | 50 | N | Biopsy | 1.87L | 2.32L | 7, 9 |
| 14 | Asthma | M | 27 | N | Biopsy | 3.75L | 5.90L | 4, 7 |
| 15 | Asthma | M | 22 | - | Biopsy | - | - | 5, 7, 9 |
| 16 | Asthma | M | 21 | N | Biopsy | 3.12L | 4.82L | 2, 3, 8, 9 |
| 17 | Asthma | M | 39 | - | Biopsy | - | - | 2, 5, 8, 9, |
| 18 | Asthma | F | 19 | - | Biopsy | 91% | - | 2, 3, 4, 8, 9 |
| 19 | Asthma | F | 27 | - | Biopsy | 2.66L | 3.83L | 2, 5, 9, 10 |
| 20 | Asthma | M | 24 | - | Biopsy | 72.8% | 77.7% | 7 |
| 21 | Asthma | F | 22 | N | Explant | - | - | 6 |
| 22 | Asthma | F | 33 | - | Explant | - | - | 6 |
| 23 | Asthma | F | 15 | - | Explant | - | - | 6 |
| 24 | Asthma | F | 34 | Y | Explant | - | - | 6 |
| 37 | Normal healthy control | M | 20 | - | Biopsy | - | - | 8 |
| 38 | Normal healthy control | F | 27 | N | Bronchoscopy | - | - | 5 |
| 39 | Normal healthy control | F | 21 | N | Bronchoscopy | - | - | 1, 2 |
| 40 | Normal healthy control | M | 69 | - | Biopsy | - | - | 1, 8 |
| 41 | Donor- trauma | - | - | - | - | - | - | 8 |
| 42 | Metastatic melanoma | F | 38 | - | Resection | - | - | 8 |
| 43 | Donor - trauma | M | 48 | N | Transplant | - | - | 1, 2, 3, 7, 8, 9, 10 |
| 44 | Normal healthy control | M | 27 | N | Bronchoscopy | - | - | 2 |
| 45 | Normal healthy control | F | 22 | - | Biopsy | - | - | 8, 9 |
| 46 | Ca | M | 61 | Ex | Resection | 112% | 118% | 4 |
| 47 | NSSCa | M | 71 | N | Resection | 93% | 89% | 5, 7, 9 |
| 48 | Ca | F | 69 | Ex | Resection | 1.21L | 1.76L | 4 |
| 49 | Squamous cell Ca | M | 66 | Ex | Resection | 1.74L | 2.90L | 4 |
| 50 | Adenocarcinoma | F | 59 | N | Resection | 111% | 106% | 5, 7, 9 |
| 51 | No diagnosis | M | 58 | N | Transplant | - | - | 5, 9, 10 |
| 52 | NSCCa | F | 58 | N | Resection | 96% | 91% | 5, 7, 9 |
| 53 | NSCCa  + malignant neoplasm | M | 59 | Ex | Resection | 76% | 73% | 2, 3, 8, 9 |
| 54 | NSCCa  + malignant neoplasm | F | 73 | Ex | Resection | 100% | 113% | 2, 9 |
| 55 | NSCCa + malignant neoplasm | F | 56 | Ex | Resection | 66% | 71% | 2, 9 |
| 56 | Squamous cell Ca  + adenocarcinoma  + malignant neoplasm | M | 75 | Ex | Resection  + bronchoscopy | 85% | 79% | 2 |
| 57 | Adenocarcinoma | F | 57 | - | resection | - | - | 5 |
| 58 | Ca | F | 50 | - | Resection | - | - | 10 |
| 59 | NSCLC + secondary malignant neoplasm + obstructive sleep apnea | M | 70 |  | Resection | 57% | 72% | 7 |
| 60 | NSCCa | M | 68 | Ex | Resection | 82% | 78% | 3, 7, 10 |
| 61 | Non-asthmatic | M | 29 | - | Explant | - | - | 6 |
| 62 | Non-asthmatic | M | 30 | - | Explant | - | - | 6 |
| 63 | Non-asthmatic | M | 35 | Y | Explant | - | - | 6 |
| 64 | Non-asthmatic | F | 57 | - | Explant | - | - | 6 |

**Experiments**: ASM gene expression: RT-PCR arrays ‘Human ECM proteins and adhesion molecules’ **(1)**, and ‘Human Angiogenesis’ **(2).** ASM-ECM composition: Total protein content **(3)**, deposited fibronectin **(4)**, deposited collagen I and III **(5)**, and *in vivo* collagen fiber organization **(6)**. ASM-ECM functional properties: HUVECs seeded on ASM-ECM were assessed for proliferation **(7)**, metabolic activity **(8)**, attachment **(9)**, and migration **(10)**.
**Abbreviations**: ASM: Airway smooth muscle, BSA: Bovine serum albumin, Ca: carcinoma, FBS: Fetal bovine serum, FEV_1_: Forced Expiratory Volume in the first second (presented as % predicted values or liters), FVC: Forced Vital Capacity (% predicted values or liters), HUVEC: Human umbilical vein endothelial cell, NCBI: National Centre for Biotechnology Information, NSSCa: non-small cell Ca, RT-PCR: Real-time PCR. Experiment numbers (shown within the brackets) are reflected in the table to illustrate cells used from *in vitro* investigations.

## Isolation and culture of primary human ASM cells and human umbilical vein endothelial cells

ASM cells were isolated from human lung tissue by macrodissection as previously described [2-4], before being used for experiments between passages 2-6.

ASM cells were grown in low glucose DMEM with 10% (v/v) FBS, 1% (v/v) Antibiotic-Antimycotic (Ab), 25mM HEPES (referred to as ‘10% FBS-DMEM culture media’)

Human umbilical vein endothelial cells (HUVECs) were isolated from umbilical cords as previously described [5], and used for experiments between passages 1-5. HUVECs were grown in M199 with 2.2g/L NaHCO_3_, 5U/mL Penicillin/Streptomycin, 2mM L-Glutamine, 20% (v/v) heat inactivated new born calf serum (NBCS), 5% (v/v) heat inactivated human serum supplemented with 45µg/mL bovine brain extract (BBE), 7.5µg/mL endothelial cell growth supplement (ECGS), or 10U/mL heparin (referred to as ‘HUVEC culture media’).

All cells (ASM and HUVEC) tested negative for the presence of Mycoplasma before use.

## Study design

This study aimed to investigate the composition and functionality of the ECM deposited by asthmatic ASM cells under non-stimulatory conditions, compared with that of non-asthmatic ASM cells. Baseline gene expression of ASM cells isolated from asthmatic or non-asthmatic individuals was assessed using real time (RT)-PCR arrays for ECM proteins, adhesion molecules, and angiogenic factors. The total mass of protein deposited, as well as the collagen I, III, and fibronectin content of the unstimulated asthmatic ASM-ECM was examined. The functionality of the asthma matrix was then assessed through examination of the behavior endothelial cells when reseeded onto the decellularized ECM generated by asthmatic ASM cells under non-stimulatory conditions. These data were compared with the cell behavioral characteristics when reseeded on the non-asthmatic ASM-ECM.

## Gene expression patterns of unstimulated non-asthmatic and asthmatic ASM cells in vitro

mRNA was isolated from the unstimulated non-asthmatic and asthmatic ASM cells after 72 hours in low glucose DMEM with 0.1% (v/v) BSA, 1% (v/v) Antibiotic-Antimycotic (Ab), 25mM HEPES (referred to as ‘0.1% BSA-DMEM quiescing media’) using a ISOLATE RNA mini Kit and a NanoDrop 1000 Spectrophotometer (NanoDrop Technologies, Inc. Wilmington, DE, USA) to determine quality and quantity before reverse transcription which was performed using MML-V as per the manufacturer’s instructions (Invitrogen). cDNA was collected from unstimulated non-asthmatic (N=3) and asthmatic (N=3) subjects, grown under non-stimulatory conditions (0.1% BSA in DMEM) for 72 hours. The samples were pooled (720ng each) to create a single non-asthmatic and asthma sample, which were loaded onto a Taqman® RT-PCR array for Human ECM & Adhesion Molecules (#4414133) as per the manufacturer’s instructions (Thermo Fisher). RT-PCR was performed using a StepOne Plus detection system and StepOne software (Life Technologies).

A second TaqMan® Array for Human Angiogenesis (#4414071) was performed using 308.6ng ASM cDNA from each of 7 non-asthmatic subjects, and 270ng ASM cDNA from 8 asthmatics, which were pooled within subject groups and added to the array plate for RT-PCR using an ABI Prism 7000 Sequence Detection System with the use of Sequence Detection Software version 1.2.3 (Thermo Fisher). Both arrays used the following cycle details: 50^o^C for 2 minutes, 95^o^C for 10 minutes, then 40 cycles of (95^o^C melting for 15 seconds, 60^o^C annealing for 1 minute and extension at 72^o^C for 1 minute). Relative abundance of gene expression was calculated using the ∆ cycle threshold (Ct) method [6], normalized to human 18S. To reduce the number of false positives introduced by an array experiment using pooled data, two housekeeping genes were used (18S and GAPDH) for normalization of the data. In a max difference analysis, a fold change of > ±2 of asthmatic vs non-asthmatic ASM cells was considered to illustrate a difference between the groups.

The results from the Taqman® RT-PCR array for Human ECM & Adhesion Molecules and TaqMan® Array for Human Angiogenesis were validated as described previously, using qRT-PCR for selected individual genes [7].

**Comparative GO term enrichment**

A Gene Ontology (GO) enrichment was performed with the R package gProfileR version 0.6.4 [8]. This package applied a correction for multiple tests designed for ontology analysis by default (i.e. **g:SCS)** [8].

To assess the GO enrichment, we follow a sampling approach. It consists in obtaining random sets of genes from the original set which consist of 121 genes. The set of genes under study (6 decreased or 17 increased) will be denoted in this section as the ‘target’ set. The main goal is to contrast the enrichment of the ‘target’ set against the enrichment of random samples of genes (of the same size than the ‘target’ set). The sampling approach can be divided in 4 steps as follows:

1. The GO enrichment analysis is performed for the ‘target’ set, and obtaining the p-value for the significant GO terms (red dots in plots of Figure 1 **B)** and **C)**).

2. 100 sets of genes having the same number of genes corresponding to the ‘target’ set (6 for decreased and 17 for increased gene set) are randomly sampled without replacement from the original set. For each of the (randomly) sampled sets, the GO enrichment analysis is performed obtaining their related GO significance

3. The GOs from random sets are matched with the significant GOs (step 2) of the ‘target’ set (step 1). In case that a significant GO of the target set (step 1) didn’t match with the GOs obtained by random (step 2) it was concluded that the sampled genes had no relationship with this biological process, and therefore the respective p-values were considered to be one.

4. All the p-values are –log_10_ transformed, thus a larger –log_10_ p-value are equivalent to a higher GO’s significance. The visual comparison shows (i) the target’s –log_10_ p-values, and (ii) the average –log_10_ p-values from the random sets with error bars denoting 2 standard errors shown only at the right side (higher values) from the mean random set p-value.

## ASM-ECM deposition and decellularization

For experimental use, non-asthmatic and asthmatic ASM cells were seeded onto tissue culture surfaces at a density of 1x10^4^ cells/cm^2^ (ASM cell viability was determined with trypan blue exclusion) and grown in 10% FBS-DMEM culture media for 72 hours. Cells were synchronized by incubation in 0.1% BSA-DMEM quiescing media for 48 hours. The ASM cells were then immersed in fresh 0.1% BSA-DMEM quiescing media for 24 hours, before being washed three times with 1x PBS and lysed with 0.016mM NH_4_OH at 37^o^C for 30-60 minutes, leaving the ASM cell-deposited ECM intact. The decellularized non-asthmatic and asthmatic ECM were washed three times with 1x PBS and either solubilized for total protein quantification in protein lysate buffer (20mM Tris-HCl, 150mM NaCl, 1mM Na_2_EDTA, 1% (v/v) Triton X-100, 10% (v/v) Glycerol, 0.1% (w/v) SDS, 0.5% (w/v) sodium deoxycholate, 1 mM PMSF, Protease Inhibitor Cocktail Set III (1:100 dilution)), or stored as an intact ECM coated onto the tissue culture surface in 1x PBS at -20^o^C.

### Total protein content of the unstimulated ASM-ECM

The solubilized ECM from non-asthmatic and asthmatic ASM cells was measured for total protein content using a Bicinchoninic Acid (BCA) kit against a BSA standard curve as per the manufacturer’s instructions (Sigma).

### Collagen and fibronectin deposition in the unstimulated ASM-ECM

In the decellularized ASM-ECM, collagen I and III content was assessed using picosirius red staining as previously described [9]. Briefly, 96 well plates previously coated with ASM-ECM were washed once with PBS-tween, picosirius red stain (50 uL/well) was then added for 1 hr at room temperature, after which excessive stain removed with 10 mM HCl (5 washes), and the stain solubilised with 0.1 M NaOH for 5 minutes. Well contents were transferred into a new 96 well plate and read at 540 nm using a Sepctromax M2 and Soft Max pro (version 4.8, Molecular Devices). A standard curve adjustment was used to calculate the concentration of protein based on the intensity of the red stain in the well. Collagen I and III as a % of total protein was determined by calculating the quantity of collagen I and III as a percentage of the total quantity of protein of ASM-ECM coated wells prepared in the same way (determined by a BCA assay [see section above]).

Fibronectin within the ECM deposited by unstimulated non-asthmatic or asthmatic ASM cells onto the surface of clear flat-bottomed 96 well tissue culture plates was quantified using a solid-phase ELISA as previously described [10].

Fibronectin and collagen I and III were quantified as % total ECM protein determined by BCA as outlined above.

### Collagen I fiber organization within in vivo ASM bundles of non-asthmatic and asthmatic individuals

Collagen I fiber organization within the ASM bundles of non-asthmatic and asthmatic airway tissue was examined by detecting the second harmonic generation (SHG) signal of collagen I as previously described [11,12]. The degree of collagen I fiber organization in a sample was determined by the forward/backward SHG ratio. Sections (30μm thick) were cut from 4 non-asthmatic and 4 asthmatic subjects using a Shandon Finesse 325 microtome (Thermo Fisher), deparaffinized and rehydrated through graded ethanol before being mounted onto glass slides with di-n-butyl phthalate in xylene (DPX). Forward and backward SHG signals within 3 randomly selected regions within the ASM bundles were detected and quantified as previously described [11,12] and the signal intensity of each was measured using Fiji software (National Institutes of Health, MD, USA) [13], with the forward/backward ratio providing a representation of collagen I organization within the non-asthmatic and asthmatic ASM bundles. The diagnosis of patients’ samples were blinded throughout the protocol and revealed only during final analysis. A Leica LAS AF software (Leica, Wetzlar, Germany) program was used to randomly select regions of the airway to be imaged and analyzed.

## The functional properties of ECM from unstimulated ASM cells: HUVEC behavior on decellularized ASM-ECM

HUVECs which had been quiescent for 36 hours in supplement-free HUVEC culture media (without additional BBE, ECGs, or heparin) and were seeded onto ECM derived from non-asthmatic and asthmatic ASM cells in the fresh supplement-free HUVEC culture media. HUVEC proliferation and metabolic activity were assessed 72 hours after seeding (1x10^5^ cells/mL) using CyQUANT and thaizolyl blue tetrazolium bromide (MTT) assays according to the manufacturers’ instructions (ThermoFisher and Sigma respectively). For assessment of attachment, HUVEC (1x10^5^ cells/mL) were plated onto ECM from unstimulated ASM cells and incubated at 37^o^C for 30 minutes. Attachment was quantified with toluidine blue staining as previously described [14] using a Spectromax M2 and Soft Max pro software (version 4.8 Molecular Devices, Sunnyvale, CA, USA). Proliferation, metabolic activity, and attachment of HUVECs to the non-asthmatic or asthmatic ASM-derived ECM was controlled to the response of HUVECs in wells which contained no ASM cells (‘no ASM-ECM’). Cell culture medium contains a multitude of proteins which readily coat tissue culture surfaces. The ‘no ASM-ECM’ control accounts for the interaction of HUVEC with these media-derived proteins. Chemotaxis of quiescent HUVEC was performed in a transwell system (8μm pore size) using VEGF-A (10ng/mL) as the chemoattractant as previously described [15]. Migrating cells were stained with toluidine blue, after fixation, and mounted onto slides in 70% glycerol/PBS. An Olympus BX60 light microscope and Olympus DP71 camera (Olympus, Tokyo, Japan) were used to photograph 5 regions on each membrane. Regions were selected at random and the number of cells per field of view (FOV) quantified manually. These assays were validated by comparing to HUVEC on tissue culture surfaces coated with 1μg/mL fibronectin or gelatin.

# Results

**Comparative GO term enrichment**

**Table S2. List of significantly enriched GOs for increased genes (**$FDR\leq0.05$**).**

| Leukocyte migration | 7.56 10^-9^ | GO:0050900 | ITGA3,CEACAM1,ITGA6,FLT1,ITGA4,IL8,S1PR1,ITGB3,PECAM1 |
| --- | --- | --- | --- |
| Anatomical structure formation involved in morphogenesis | 1.55 10^-8^ | GO:0048646 | ITGA3,CEACAM1,ITGA6,FLT1,ITGA4,ITGA7,HEY1,IL8,S1PR1,ITGB3,PECAM1 |
| Integrin cell surface interactions | 3.55 10^-8^ | REAC:R-HSA-216083 | ITGA3,ITGA6,ITGA4,ITGA7,ITGB3,PECAM1 |
| Integrin complex | 3.71 10^-8^ | GO:0008305 | ITGA3,ITGA6,ITGA4,ITGA7,ITGB3 |
| Regulation of cell migration | 4.23 10^-8^ | GO:0030334 | ITGA3,CEACAM1,ITGA6,FLT1,ITGA4,MMP10,IL8,S1PR1,ITGB3,PECAM1 |
| Protein complex involved in cell adhesion | 5.88 10^-8^ | GO:0098636 | ITGA3,ITGA6,ITGA4,ITGA7,ITGB3 |
| Regulation of cell motility | 7.71 10^-8^ | GO:2000145 | ITGA3,CEACAM1,ITGA6,FLT1,ITGA4,MMP10,IL8,S1PR1,ITGB3,PECAM1 |
| Integrin-mediated signaling pathway | 1.01 10^-7^ | GO:0007229 | ITGA3,CEACAM1,ITGA6,ITGA4,ITGA7,ITGB3 |
| Regulation of locomotion | 1.64 10^-7^ | GO:0040012 | ITGA3,CEACAM1,ITGA6,FLT1,ITGA4,MMP10,IL8,S1PR1,ITGB3,PECAM1 |
| Regulation of cellular component movement | 1.85 10^-7^ | GO:0051270 | ITGA3,CEACAM1,ITGA6,FLT1,ITGA4,MMP10,IL8,S1PR1,ITGB3,PECAM1 |
| Extracellular matrix organization^*^ | 2.34 10^-7^ | REAC:R-HSA-1474244 | ITGA3,CEACAM1,ITGA6,ITGA4,ITGA7,MMP10,ITGB3,PECAM1 |
| Receptor complex | 1.97 10^-6^ | GO:0043235 | ITGA3,CEACAM1,ITGA6,FLT1,ITGA4,ITGA7,ITGB3 |
| Extracellular matrix organization^*^ | 2.24 10^-6^ | GO:0030198 | ITGA3,ITGA6,ITGA4,ITGA7,MMP10,ITGB3,PECAM1 |
| Plasma membrane receptor complex | 2.69 10^-6^ | GO:0098802 | ITGA3,CEACAM1,ITGA6,ITGA4,ITGA7,ITGB3 |
| Arrhythmogenic right ventricular cardiomyopathy (ARVC) | 3.30 10^-6^ | KEGG:05412 | ITGA3,ITGA6,ITGA4,ITGA7,ITGB3 |
| Cell adhesion | 3.34 10^-6^ | GO:0007155 | ITGA3,CEACAM1,ITGA6,ITGA4,ITGA7,IL8,S1PR1,IFNB1,ITGB3,PECAM1 |
| Biological adhesion | 3.54 10^-6^ | GO:0022610 | ITGA3,CEACAM1,ITGA6,ITGA4,ITGA7,IL8,S1PR1,IFNB1,ITGB3,PECAM1 |
| Extracellular structure organization^*^ | 6.25 10^-6^ | GO:0043062 | ITGA3,ITGA6,ITGA4,ITGA7,MMP10,ITGB3,PECAM1 |
| ECM-receptor interaction^*^ | 6.38 10^-6^ | KEGG:04512 | ITGA3,ITGA6,ITGA4,ITGA7,ITGB3 |
| Hypertrophic cardiomyopathy (HCM) | 6.79 10^-6^ | KEGG:05410 | ITGA3,ITGA6,ITGA4,ITGA7,ITGB3 |
| Cell migration | 7.85 10^-6^ | GO:0016477 | ITGA3,CEACAM1,ITGA6,FLT1,ITGA4,MMP10,IL8,S1PR1,ITGB3,PECAM1 |
| Cell-matrix adhesion | 8.19 10^-6^ | GO:0007160 | ITGA3,ITGA6,ITGA4,ITGA7,ITGB3,PECAM1 |
| Dilated cardiomyopathy (DCM) | 9.65 10^-6^ | KEGG:05414 | ITGA3,ITGA6,ITGA4,ITGA7,ITGB3 |
| Tube development | 1.36 10^-5^ | GO:0035295 | ITGA3,CEACAM1,ITGA6,FLT1,HEY1,IL8,S1PR1,ITGB3,PECAM1 |
| Human papillomavirus infection | 1.57 10^-5^ | KEGG:05165 | ITGA3,ITGA6,ITGA4,ITGA7,HEY1,IFNB1,ITGB3 |
| Focal adhesion | 1.68 10^-5^ | KEGG:04510 | ITGA3,ITGA6,FLT1,ITGA4,ITGA7,ITGB3 |
| Cell surface receptor signaling pathway | 1.8 10^-5^ | GO:0007166 | ITGA3,CEACAM1,ITGA6,FLT1,ITGA4,ITGA7,HEY1,IL8,S1PR1,IFNB1,TNFSF15,ITGB3 |
| Localization of cell | 1.94 10^-5^ | GO:0051674 | ITGA3,CEACAM1,ITGA6,FLT1,ITGA4,MMP10,IL8,S1PR1,ITGB3,PECAM1 |
| Cell motility | 1.94 10^-5^ | GO:0048870 | ITGA3,CEACAM1,ITGA6,FLT1,ITGA4,MMP10,IL8,S1PR1,ITGB3,PECAM1 |
| PI3K-Akt signaling pathway | 2.07 10^-5^ | KEGG:04151 | ITGA3,ITGA6,FLT1,ITGA4,ITGA7,IFNB1,ITGB3 |
| Cell surface interactions at the vascular wall | 2.18 10^-5^ | REAC:R-HSA-202733 | ITGA3,CEACAM1,ITGA6,ITGA4,ITGB3,PECAM1 |
| Cell surface | 2.57 10^-5^ | GO:0009986 | ITGA3,CEACAM1,ITGA6,ITGA4,ITGA7,S1PR1,ITGB3,PECAM1 |
| Positive regulation of cell migration | 3.6 10^-5^ | GO:0030335 | ITGA3,ITGA6,FLT1,ITGA4,IL8,S1PR1,ITGB3 |
| Positive regulation of cell motility | 4.54 10^-5^ | GO:2000147 | ITGA3,ITGA6,FLT1,ITGA4,IL8,S1PR1,ITGB3 |
| Positive regulation of cellular component movement | 5.6 10^-5^ | GO:0051272 | ITGA3,ITGA6,FLT1,ITGA4,IL8,S1PR1,ITGB3 |
| Positive regulation of locomotion | 6.79 10^-5^ | GO:0040017 | ITGA3,ITGA6,FLT1,ITGA4,IL8,S1PR1,ITGB3 |
| Locomotion | 7.18 10^-5^ | GO:0040011 | ITGA3,CEACAM1,ITGA6,FLT1,ITGA4,MMP10,IL8,S1PR1,ITGB3,PECAM1 |
| Cell-substrate adhesion | 9.46 10^-5^ | GO:0031589 | ITGA3,ITGA6,ITGA4,ITGA7,ITGB3,PECAM1 |
| Angiogenesis | 9.81 10^-5^ | GO:0001525 | CEACAM1,FLT1,HEY1,IL8,S1PR1,ITGB3,PECAM1 |
| Anatomical structure morphogenesis * | 0.000105 | GO:0009653 | ITGA3,CEACAM1,ITGA6,FLT1,ITGA4,ITGA7,HEY1,IL8,S1PR1,ITGB3,PECAM1 |
| Regulation of cell adhesion | 0.000211 | GO:0030155 | ITGA3,CEACAM1,ITGA6,ITGA4,IL8,S1PR1,IFNB1 |
| Movement of cell or subcellular component | 0.000215 | GO:0006928 | ITGA3,CEACAM1,ITGA6,FLT1,ITGA4,MMP10,IL8,S1PR1,ITGB3,PECAM1 |
| Blood vessel morphogenesis | 0.000266 | GO:0048514 | CEACAM1,FLT1,HEY1,IL8,S1PR1,ITGB3,PECAM1 |
| Circulatory system development | 0.000498 | GO:0072359 | ITGA3,CEACAM1,FLT1,HEY1,IL8,S1PR1,ITGB3,PECAM1 |
| Immune system process | 0.000562 | GO:0002376 | ITGA3,CEACAM1,ITGA6,FLT1,ITGA4,IL8,S1PR1,IFNB1,TNFSF15,ITGB3,PECAM1 |
| Blood vessel development | 0.000571 | GO:0001568 | CEACAM1,FLT1,HEY1,IL8,S1PR1,ITGB3,PECAM1 |
| Intrinsic component of plasma membrane | 0.000578 | GO:0031226 | ITGA3,CEACAM1,ITGA6,FLT1,ITGA4,ITGA7,S1PR1,TNFSF15,ITGB3 |
| Hematopoietic cell lineage | 0.000633 | KEGG:04640 | ITGA3,ITGA6,ITGA4,ITGB3 |
| Regulation of actin cytoskeleton | 0.000732 | KEGG:04810 | ITGA3,ITGA6,ITGA4,ITGA7,ITGB3 |
| Cell-cell adhesion | 0.000766 | GO:0098609 | CEACAM1,ITGA6,ITGA4,ITGA7,IFNB1,ITGB3,PECAM1 |
| Vasculature development | 0.000786 | GO:0001944 | CEACAM1,FLT1,HEY1,IL8,S1PR1,ITGB3,PECAM1 |
| Regulation of response to stimulus | 0.000844 | GO:0048583 | ITGA3,CEACAM1,ITGA6,FLT1,ITGA4,HEY1,IL8,S1PR1,IFNB1,TNFSF15,ITGB3,PECAM1 |
| Laminin interactions | 0.000847 | REAC:R-HSA-3000157 | ITGA3,ITGA6,ITGA7 |
| Cardiovascular system development | 0.00085 | GO:0072358 | CEACAM1,FLT1,HEY1,IL8,S1PR1,ITGB3,PECAM1 |
| Fibronectin binding | 0.00124 | GO:0001968 | ITGA3,ITGA4,ITGB3 |
| Endothelial cell differentiation | 0.00147 | GO:0045446 | CEACAM1,HEY1,S1PR1,PECAM1 |
| Adherens junction | 0.0015 | GO:0005912 | ITGA3,CEACAM1,ITGA6,FLT1,ITGA4,ITGB3 |
| Anchoring junction | 0.00181 | GO:0070161 | ITGA3,CEACAM1,ITGA6,FLT1,ITGA4,ITGB3 |
| Cellular response to organic substance | 0.00196 | GO:0071310 | ITGA3,CEACAM1,ITGA6,FLT1,ITGA4,IL8,S1PR1,IFNB1,TNFSF15,ITGB3 |
| Tissue development | 0.00206 | GO:0009888 | ITGA3,CEACAM1,ITGA6,ITGA4,ITGA7,HEY1,S1PR1,ITGB3,PECAM1 |
| Tube morphogenesis | 0.00207 | GO:0035239 | CEACAM1,FLT1,HEY1,IL8,S1PR1,ITGB3,PECAM1 |
| Plasma membrane protein complex | 0.00211 | GO:0098797 | ITGA3,CEACAM1,ITGA6,ITGA4,ITGA7,ITGB3 |
| Hemostasis | 0.00221 | REAC:R-HSA-109582 | ITGA3,CEACAM1,ITGA6,ITGA4,IFNB1,ITGB3,PECAM1 |
| Regulation of localization | 0.00251 | GO:0032879 | ITGA3,CEACAM1,ITGA6,FLT1,ITGA4,MMP10,IL8,S1PR1,ITGB3,PECAM1 |
| Endothelium development | 0.00266 | GO:0003158 | CEACAM1,HEY1,S1PR1,PECAM1 |
| Formation of primary germ layer | 0.00293 | GO:0001704 | ITGA3,ITGA4,ITGA7,ITGB3 |
| Plasma membrane part | 0.00319 | GO:0044459 | ITGA3,CEACAM1,ITGA6,FLT1,ITGA4,ITGA7,S1PR1,TNFSF15,ITGB3,PECAM1 |
| Extracellular region part | 0.00323 | GO:0044421 | ITGA3,CEACAM1,ITGA6,FLT1,ITGA4,MMP10,IL8,IFNB1,TNFSF15,ITGB3,PECAM1 |
| Embryo development | 0.00342 | GO:0009790 | ITGA3,FLT1,ITGA4,ITGA7,HEY1,IL8,ITGB3 |
| Signal transduction | 0.00433 | GO:0007165 | ITGA3,CEACAM1,ITGA6,FLT1,ITGA4,ITGA7,HEY1,IL8,S1PR1,IFNB1,TNFSF15,ITGB3,PECAM1 |
| System development | 0.00449 | GO:0048731 | ITGA3,CEACAM1,ITGA6,FLT1,ITGA4,ITGA7,HEY1,IL8,S1PR1,IFNB1,ITGB3,PECAM1 |
| Diapedesis | 0.00532 | GO:0050904 | ITGA4,PECAM1 |
| Integral component of plasma membrane | 0.00655 | GO:0005887 | ITGA3,CEACAM1,ITGA6,FLT1,ITGA4,ITGA7,TNFSF15,ITGB3 |
| C-X3-C chemokine binding | 0.00885 | GO:0019960 | ITGA4,ITGB3 |
| Neuregulin binding | 0.00885 | GO:0038132 | ITGA6,ITGB3 |
| Positive regulation of cellular process | 0.00987 | GO:0048522 | ITGA3,CEACAM1,ITGA6,FLT1,ITGA4,HEY1,IL8,S1PR1,IFNB1,TNFSF15,ITGB3,PECAM1 |
| Cellular response to chemical stimulus | 0.0107 | GO:0070887 | ITGA3,CEACAM1,ITGA6,FLT1,ITGA4,IL8,S1PR1,IFNB1,TNFSF15,ITGB3 |
| Focal adhesion | 0.0107 | GO:0005925 | ITGA3,ITGA6,FLT1,ITGA4,ITGB3 |
| Cell-substrate adherens junction | 0.0111 | GO:0005924 | ITGA3,ITGA6,FLT1,ITGA4,ITGB3 |
| Signaling | 0.0114 | GO:0023052 | ITGA3,CEACAM1,ITGA6,FLT1,ITGA4,ITGA7,HEY1,IL8,S1PR1,IFNB1,TNFSF15,ITGB3,PECAM1 |
| Regulation of molecular function | 0.0115 | GO:0065009 | CEACAM1, ITGA6,FLT1,ITGA4,HEY1,IL8,S1PR1,IFNB1,TNFSF15,ITGB3 |
| Regulation of signal transduction | 0.0116 | GO:0009966 | ITGA3,CEACAM1,ITGA6,FLT1,HEY1,IL8,IFNB1,TNFSF15,ITGB3,PECAM1 |
| Heterotypic cell-cell adhesion | 0.0118 | GO:0034113 | ITGA4,ITGA7,ITGB3 |
| Cell-substrate junction | 0.0118 | GO:0030055 | ITGA3,ITGA6,FLT1,ITGA4,ITGB3 |
| Cell differentiation | 0.0121 | GO:0030154 | ITGA3,CEACAM1,ITGA6,FLT1,ITGA4,ITGA7,HEY1,S1PR1,IFNB1,ITGB3,PECAM1 |
| Cell communication | 0.0124 | GO:0007154 | ITGA3,CEACAM1,ITGA6,FLT1,ITGA4,ITGA7,HEY1,IL8,S1PR1,IFNB1,TNFSF15,ITGB3,PECAM1 |
| Extracellular matrix binding | 0.0125 | GO:0050840 | ITGA3,ITGA6,ITGB3 |
| Response to organic substance | 0.0129 | GO:0010033 | ITGA3,CEACAM1,ITGA6,FLT1,ITGA4,IL8,S1PR1,IFNB1,TNFSF15,ITGB3 |
| Gastrulation | 0.0135 | GO:0007369 | ITGA3,ITGA4,ITGA7,ITGB3 |
| Ameboidal-type cell migration | 0.0157 | GO:0001667 | ITGA3,CEACAM1,ITGA4,ITGB3,PECAM1 |
| Multicellular organism development | 0.016 | GO:0007275 | ITGA3,CEACAM1,ITGA6,FLT1,ITGA4,ITGA7,HEY1,IL8,S1PR1,IFNB1,ITGB3,PECAM1 |
| Actin-based cell projection | 0.0166 | GO:0098858 | ITGA3,CEACAM1,ITGA6,ITGB3 |
| Cell junction | 0.0189 | GO:0030054 | ITGA3,CEACAM1,ITGA6,FLT1,ITGA4,ITGB3,PECAM1 |
| Cellular developmental process | 0.019 | GO:0048869 | ITGA3,CEACAM1,ITGA6,FLT1,ITGA4,ITGA7,HEY1,S1PR1,IFNB1,ITGB3,PECAM1 |
| Extracellular space | 0.023 | GO:0005615 | ITGA3,CEACAM1,FLT1,ITGA4,MMP10,IL8,IFNB1,TNFSF15,ITGB3,PECAM1 |
| Regulation of developmental process | 0.0231 | GO:0050793 | ITGA3,CEACAM1,FLT1,ITGA7,HEY1,IL8,S1PR1,IFNB1,ITGB3 |
| Animal organ development | 0.0259 | GO:0048513 | ITGA3,CEACAM1,ITGA6,ITGA4,ITGA7,HEY1,IL8,S1PR1,IFNB1,PECAM1 |
| Regulation of cell communication | 0.0272 | GO:0010646 | ITGA3,CEACAM1,ITGA6,FLT1,HEY1,IL8,IFNB1,TNFSF15,ITGB3,PECAM1 |
| PECAM1 interactions | 0.028 | REAC:R-HSA-210990 | ITGB3,PECAM1 |
| Extracellular region | 0.0292 | GO:0005576 | ITGA3,CEACAM1,ITGA6,FLT1,ITGA4,MMP10,IL8,IFNB1,TNFSF15,ITGB3,PECAM1 |
| Regulation of signaling | 0.0299 | GO:0023051 | ITGA3,CEACAM1,ITGA6,FLT1,HEY1,IL8,IFNB1,TNFSF15,ITGB3,PECAM1 |
| Positive regulation of biological process | 0.0394 | GO:0048518 | ITGA3,CEACAM1,ITGA6,FLT1,ITGA4,HEY1,IL8,S1PR1,IFNB1,TNFSF15,ITGB3,PECAM1 |
| Anatomical structure development | 0.0417 | GO:0048856 | ITGA3,CEACAM1,ITGA6,FLT1,ITGA4,ITGA7,HEY1,IL8,S1PR1,IFNB1,ITGB3,PECAM1 |

* Non-significant pathway in GO enrcihment analysis using sets of randomly sampled genes. This sets have the same number of genes as the increased genes from the original analyzis (17 genes). Sampling without replacement were taken from the complete list of genes (121 genes). FDR: false discovery rate

**Table S3. List of significantly enriched GOs for the decreased genes (**$FDR\leq0.05$**).**

| **Term ID** | ***p-value*** | **Term name** | **Intersection** |
| --- | --- | --- | --- |
| **Integrin cell surface interactions** | ***3.58 10-06*** | **REAC:R-HSA-216083** | **ITGA8, VTN, KDR, VCAM1** |
| **ECM organization** | ***1.05 10-05*** | **GO:0030198** | **ITGA8, VTN, KDR, VCAM1, COL14A1** |
| **ECM organization** | ***1.70 10-05*** | **REAC:R-HSA-1474244** | **ITGA8, VTN, KDR, VCAM1, COL14A1** |
| **Extracellular structure organization** | ***2.21 10-05*** | **GO:0043062** | **ITGA8, VTN, KDR, VCAM1, COL14A1** |
| **Cell-matrix adhesion** | ***0.00026*** | **GO:0007160** | **ITGA8, VTN, KDR, VCAM1** |
| **Cell-substrate adhesion** | ***0.00135*** | **GO:0031589** | **ITGA8, VTN, KDR, VCAM1** |
| **Integrin binding** | ***0.00590*** | **GO:0005178** | **VTN, KDR, VCAM1** |
|  |  |  |  |
| **Cell adhesion** | ***0.0112*** | **GO:0007155** | **ITGA8, VTN, KDR, VCAM1, COL14A1** |
| **Biological adhesion** | ***0.0115*** | **GO:0022610** | **ITGA8, VTN, KDR, VCAM1, COL14A1** |
| **Focal adhesion** | ***0.0165*** | **KEGG:04510** | **ITGA8, VTN, KDR** |
| **Proteoglycans in cancer** | ***0.0165*** | **KEGG:05205** | **TIMP3, VTN, KDR** |
| **Signaling mediated From intracellular calcium** | ***0.0192*** | **GO:0035584** | **KDR, VCAM1** |
| **Molecules associated with elastic fibres** | ***0.0202*** | **REAC:R-HSA-2129379** | **ITGA8, VTN** |
| **Cellular response to growth factor stimulus** | ***0.0221*** | **GO:0071363** | **ITGA8, VTN, KDR, VCAM1** |
| **Response to growth factor** | ***0.0379*** | **GO:0070848** | **ITGA8, VTN, KDR, VCAM1** |
| **Elastic fibre formation** | ***0.0322*** | **REAC:R-HSA-1566948** | **ITGA8, VTN** |

**Abbreviations**: FDR: false discovery rate.

**Validation of RT-PCR Array gene expression patterns**


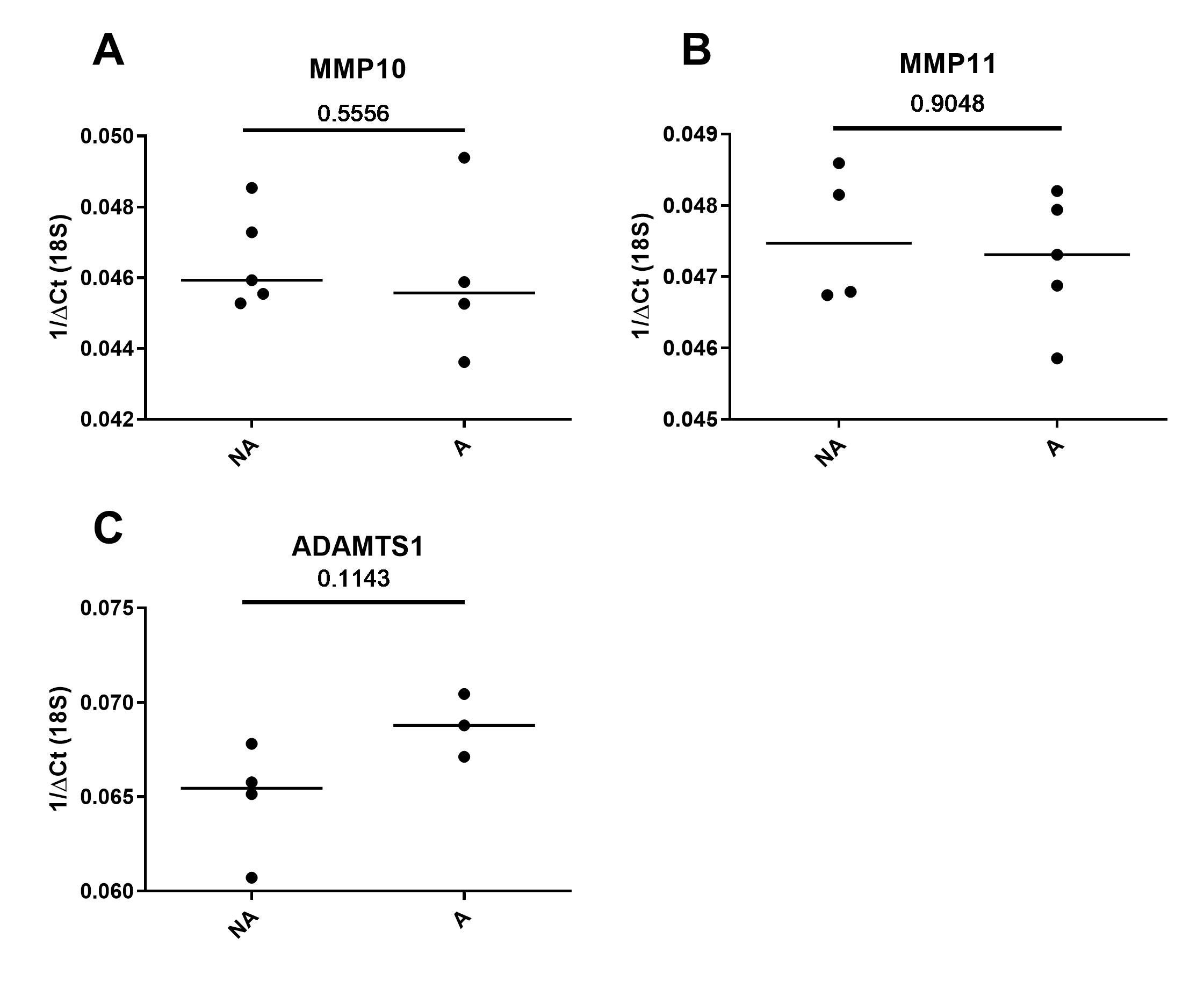


Figure S1 Validation of gene expression in asthmatic and non-asthmatic airway smooth muscle cells. mRNA was isolated from unstimulated non-asthmatic (NA, n=3-7) and asthmatic (A, n=3-5) under non-stimulatory conditions (0.1% BSA in quiescing media) for 72 hours. Gene expression levels were measured using qRT_PCR. Differences in gene expression were tested using Mann Whitney U test.

# References

1. **Baraket M, Oliver BG, Burgess JK*, et al.*** Is low dose inhaled corticosteroid therapy as effective for inflammation and remodeling in asthma? A randomized, parallel group study. *Respiratory research*. 2012; 13: 11.

2. **Faiz A, Donovan C, Nieuwenhuis MA*, et al.*** Latrophilin receptors: novel bronchodilator targets in asthma. *Thorax*. 2017; 72: 74-82.

3. **Faiz A, Weckmann M, Tasena H*, et al.*** Profiling of healthy and asthmatic airway smooth muscle cells following IL-1beta treatment: a novel role for CCL20 in chronic mucus hyper-secretion. *Eur Respir J*. 2018.

4. **Johnson PRA, Roth M, Tamm M*, et al.*** Airway smooth muscle cell proliferation is increased in asthma. *Am J Respir Crit Care Med*. 2001; 164: 474-7.

5. **Hotchkiss KA, Ashton AW, Klein RS*, et al.*** Mechanisms by which tumor cells and monocytes expressing the angiogenic factor thymidine phosphorylase mediate human endothelial cell migration. *Cancer Res*. 2003; 63: 527-33.

6. **Livak KJ, Schmittgen TD.** Analysis of relative gene expression data using real-time quantitative PCR and the 2(-Delta Delta C(T)) Method. *Methods*. 2001; 25: 402-8.

7. **Harkness LM, Weckmann M, Kopp M*, et al.*** Tumstatin regulates the angiogenic and inflammatory potential of airway smooth muscle extracellular matrix. *J Cell Mol Med*. 2017.

8. **Reimand J, Arak T, Adler P*, et al.*** g:Profiler-a web server for functional interpretation of gene lists (2016 update). *Nucleic Acids Res*. 2016; 44: W83-9.

9. **Walsh BJ, Thornton SC, Penny R, Breit SN.** Microplate reader-based quantitation of collagens. *Anal Biochem*. 1992; 203: 187-90.

10. **Krimmer DI, Burgess JK, Wooi TK*, et al.*** Matrix proteins from smoke-exposed fibroblasts are pro-proliferative. *American journal of respiratory cell and molecular biology*. 2012; 46: 34-9.

11. **Tjin G, Xu P, Kable SH*, et al.*** Quantification of collagen I in airway tissues using second harmonic generation. *J Biomed Opt*. 2014; 19: 36005.

12. **Tjin G, White ES, Faiz A*, et al.*** Lysyl oxidases regulate fibrillar collagen remodelling in idiopathic pulmonary fibrosis. *Dis Model Mech*. 2017; 10: 1301-12.

13. **Schindelin J, Arganda-Carreras I, Frise E*, et al.*** Fiji: an open-source platform for biological-image analysis. *Nat Methods*. 2012; 9: 676-82.

14. **Moir LM, Black JL, Krymskaya VP.** TSC2 modulates cell adhesion and migration via integrin-alpha1beta1. *Am J Physiol Lung Cell Mol Physiol*. 2012; 303: L703-10.

15. **Ashton AW, Ware JA.** Thromboxane A2 receptor signaling inhibits vascular endothelial growth factor-induced endothelial cell differentiation and migration. *Circ Res*. 2004; 95: 372-9.
